# Hydrogen sulfide signaling in plant response to temperature stress

**DOI:** 10.3389/fpls.2024.1337250

**Published:** 2024-03-13

**Authors:** Zhong-Guang Li, Jue-Rui Fang, Su-Jie Bai

**Affiliations:** ^1^ School of Life Sciences, Yunnan Normal University, Kunming, China; ^2^ Engineering Research Center of Sustainable Development and Utilization of Biomass Energy, Ministry of Education, Kunming, China; ^3^ Key Laboratory of Biomass Energy and Environmental Biotechnology, Yunnan Province, Yunnan Normal University, Kunming, China

**Keywords:** hydrogen sulfide, high and low temperature, protein persulfidation, temperature stress, stress tolerance

## Abstract

For the past 300 years, hydrogen sulfide (H_2_S) has been considered a toxic gas. Nowadays, it has been found to be a novel signaling molecule in plants involved in the regulation of cellular metabolism, seed germination, plant growth, development, and response to environmental stresses, including high temperature (HT) and low temperature (LT). As a signaling molecule, H_2_S can be actively synthesized and degraded in the cytosol, chloroplasts, and mitochondria of plant cells by enzymatic and non-enzymatic pathways to maintain homeostasis. To date, plant receptors for H_2_S have not been found. It usually exerts physiological functions through the persulfidation of target proteins. In the past 10 years, H_2_S signaling in plants has gained much attention. Therefore, in this review, based on that same attention, H_2_S homeostasis, protein persulfidation, and the signaling role of H_2_S in plant response to HT and LT stress were summarized. Also, the common mechanisms of H_2_S-induced HT and LT tolerance in plants were updated. These mechanisms involve restoration of biomembrane integrity, synthesis of stress proteins, enhancement of the antioxidant system and methylglyoxal (MG) detoxification system, improvement of the water homeostasis system, and reestablishment of Ca^2+^ homeostasis and acid-base balance. These updates lay the foundation for further understanding the physiological functions of H_2_S and acquiring temperature-stress-resistant crops to develop sustainable food and agriculture.

## Introduction

Hydrogen sulfide (H_2_S) has long been deemed a toxic gas since its discovery as a sewer gas in the 1700s (Wang, 2012; Modolo and da-Silva, 2023). H_2_S has a high affinity for heme-containing proteins, such as hemoglobin, myoglobin, cytochrome oxidase, catalase (CAT), and peroxidase (POD), and acts through protein persulfidation. Persulfidation can change the conformation and activity of proteins, leading to disruption of cellular metabolism, and even cell death (Li et al., 2016a; Liu et al., 2021; Li et al., 2022; Yang et al., 2022; Huang and Xie, 2023). At the end of the Permian period, a large amount of H_2_S was accumulated on earth due to volcano eruptions, which caused a rapid decrease of oxygen (O_2_) in seawater. This decrease promoted the mass proliferation of anaerobic green sulfur bacteria, which used sulfate (SO_4_^2-^) instead of O_2_ for respiration and released a mass of H_2_S. The released H_2_S entered the atmosphere and land, therefore leading to the extinction of 95% of marine species and 70% of terrestrial vertebrates (Wang, 2012; Li et al., 2016a, Li et al., 2022). In plants, H_2_S can result in necrosis, shedding, and growth inhibition of leaves and can inhibit the uptake of mineral elements, such as phosphate, by roots (Wang, 2012; Liu et al., 2021; Yang et al., 2022; Huang and Xie, 2023). Recently, Zhang et al. (2017) reported that H_2_S could inhibit taproot growth and regulate root system architecture in *Arabidopsis* plants by inducing reactive oxygen species (ROS) and nitric oxide (NO) to generate oxidative and nitrosative stress.

Promisingly, H_2_S has now been found to be a novel signaling molecule involved in many physiological processes in plants. These processes include cell metabolism and division, seed germination and dormancy, plant growth and development, organ maturation and senescence, and plant response to environmental stresses, including high temperature (HT) and low temperature (LT) (Li et al., 2016a; Liu et al., 2021; Wang et al., 2021; Yang et al., 2022; Huang et al., 2023). H_2_S, similar to other signaling molecules, such as calcium ion (Ca^2+^), hydrogen peroxide (H_2_O_2_), NO, methylglyoxal (MG), and glutamic acid, has a dual role, namely as a signaling molecule at low concentrations and as a toxic agent at high concentrations (Qiu et al., 2020; Iqbal et al., 2022; Pande et al., 2022; Huang and Xie, 2023; Mhamdi, 2023). Therefore, it must maintain homeostasis in plant cells under normal physiological and unfavorable environmental conditions. H_2_S homeostasis can be tightly regulated by enzymatic and non-enzymatic pathways, which are described as follows (Li et al., 2016a; Liu et al., 2021; Moseler et al., 2021; Li et al., 2022; Citi et al., 2023; Pedre et al., 2023).

In the past decade, H_2_S, as a pleiotropic signaling molecule in plants has gained much attention, especially in its metabolism and physiological function under HT and LT stress conditions (Li et al., 2016a; Liu et al., 2021; Moseler et al., 2021; Wang et al., 2021; Yang et al., 2022; Citi et al., 2023; Huang and Xie, 2023; Pedre et al., 2023). Therefore, in this review, based on that same attention, the enzymatic and non-enzymatic pathways of H_2_S homeostasis, protein persulfidation, and the signaling role of H_2_S in plant response to HT and LT stress were updated. This laid the groundwork for further understanding the physiological function of H_2_S and the acquisition of temperature-stress-resistant crops to develop sustainable food and agriculture.

## H_2_S homeostasis

**Figure 1:**
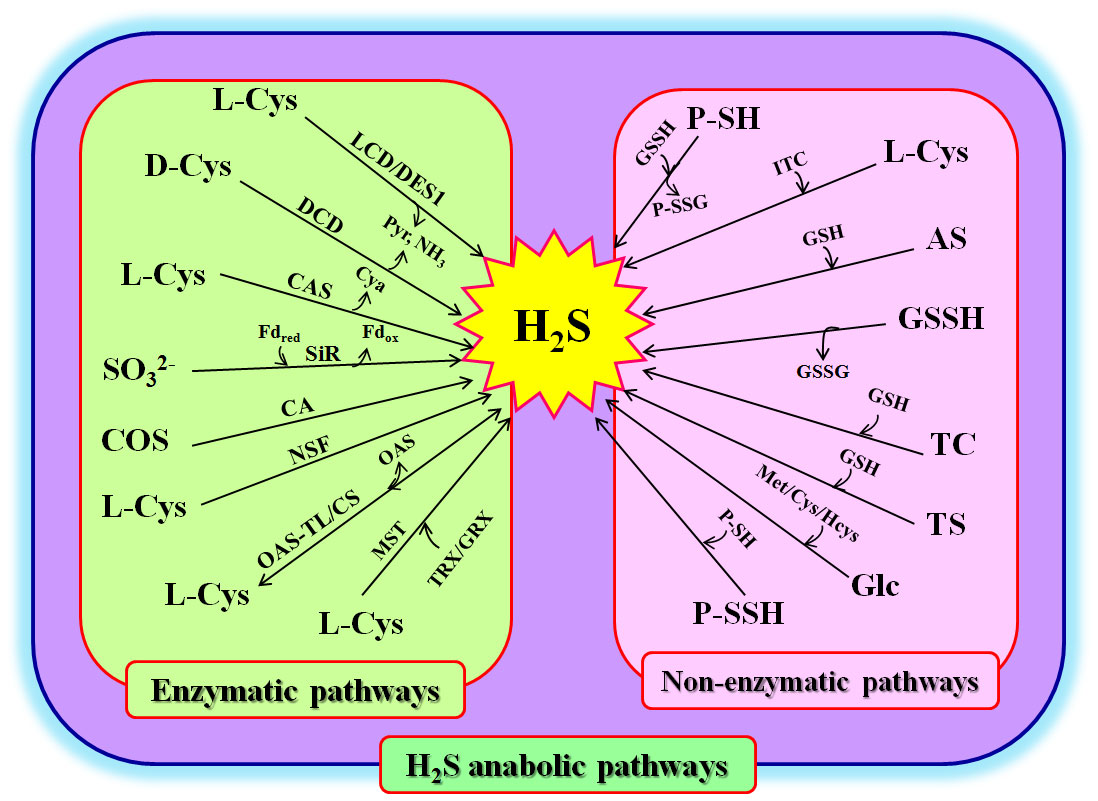
Hydrogen sulfide (H_2_S) anabolism in plants. AS, allyl sulfur; CA, carbonic anhydrase; L-Cys, L-cysteine; D-Cys, D-cysteine; CAS, β-cyanoalanine synthase; Cya, β-cyanoalanine; COS, carbonyl sulfur; CS, cysteine synthetase; LCD, L-cysteine desulfhydrase; DCD, D-cysteine desulfhydrase; DES1, cysteine desulfhydrase1; Fd_red_, reduced ferredoxin; Fd_ox_, oxidized ferredoxin; Glc, glucose; GRX, glutaredoxin; GSH, glutathione; GSSH, glutathione persulfide; Hcys, homocysteine; ITC, thiocysteine; Met, methionine; MST, 3-mercaptopyruvate sulfur transferase; NH_3_, ammonia; NSF, nitrogenase Fe-S cluster; OAS-TL, O-acetylserine (thiol) lyase; P-SH, protein thiols; P-SSH, protein persulfidation P-SSG, glutathionylated proteins; Pyr, pyruvate; SiR, sulfite reductase; TC, isothiocyanate; TRX, thioredoxin; TS, thiosulfate.

Because H_2_S is a toxic agent at high concentrations, it must maintain homeostasis in plant cells. Its homeostasis is tightly controlled by biosynthesis and degradation via enzymatic and non-enzymatic pathways (**Figure 1**). The mechanisms controlling H_2_S homeostasis in plant cells are detailed below.

### Enzymatic pathways for H_2_S biosynthesis

Involvement in multiple pathways is one of the characteristics of signaling molecules in plants (Liu et al., 2021; Wang et al., 2021). In general, H_2_S can be biosynthesized via enzymatic pathways in the cytosol, chloroplasts, and mitochondria of plants. These enzymes include L-cysteine (L-Cys) desulfhydrase (LCD), D-cysteine (D-Cys) desulfhydrase (DCD), O-acetyl-serine (thiol) lyase (OAS-TL), sulfite reductase (SiR), β-cyanoalanine synthase (CAS), and nitrogenase Fe-S cluster-like (NFS) (Liu et al., 2021; Wang et al., 2021; Huang and Xie, 2023) (**Figure 1**). In the cytosol, H_2_S can be produced from L-Cys or D-Cys under the catalysis of LCD or DCD, meanwhile releasing ammonia (NH_3_) and pyruvate (Pyr). In addition, L-Cys desulfhydrase 1 (DES1), an LCD homolog, can catalyze L-Cys to H_2_S, resulting in the production of NH_3_ and Pyr. H_2_S can also be used to synthesize L-Cys (by OAS-TL, also known as L-Cys synthetase, CS), whose reverse reaction can produce H_2_S and O-acetyl-serine (OAS) (Li et al., 2016a; Yang et al., 2022; Huang and Xie, 2023). In chloroplasts, sulfite (SO_3_^2-^), which is reduced from SO_4_^2-^, can be converted to H_2_S by SiR using ferredoxin (Fd_red_) as a cofactor. Similarly, NFS can use L-Cys to synthesize H_2_S (Aroca et al., 2020; Saud et al., 2022; Huang and Xie, 2023). In mitochondria, H_2_S can be formed under the catalysis of CAS using hydrogen cyanide (HCN) and L-Cys as substrates, along with the release of cyanoalanine (Cya) and the scavenging of HCN. Analogously, L-Cys can synthesize H_2_S via the catalysis of NFS, similar to the action of NFS in chloroplasts. OAS-TL can also catalyze L-Cys to H_2_S in mitochondria (Liu et al., 2021; Yang et al., 2022; Huang and Xie, 2023) (**Figure 1**).

In addition, carbonyl sulfide (COS), as an atmospheric trace gas, can enter the plant cells through stomata in association with carbon dioxide (CO_2_). Under the catalysis of carbonic anhydrase (CA), COS can be converted to H_2_S (Stimler et al., 2010; Li et al., 2022; Citi et al., 2023) (**Figure 1**). In addition, in *Arabidopsis* plants, 3-mercaptopyruvate sulfur transferase (MST) can be persulfidated by 3-mercaptopyruvate to form persulfidated MST (MST-SSH). The MST-SSH can release H_2_S by interacting with either thioredoxin (TRX) or glutaredoxin (GRX). Therefore, the redox systems, TRX and GRX, can not only regulate the activity of MST, but are also one of the biosynthetic pathways of H_2_S in plants (Moseler et al., 2021). Recently, MST was found to directly lead to the persulfidation of target proteins, it is considered a protein persulfidase (Pedre et al., 2023) (**Figure 1**).

### Non-enzymatic pathways for H_2_S production

As mentioned above, MST is a protein persulfidase, and the sulfur in MST-SSH can be transferred to other proteins containing dithiols (two sulfydryl groups) to form protein persulfides (P-SSH). The P-SSH can be oxidized by oxidants, releasing H_2_S (Pedre et al., 2023) (**Figure 1**). MST-SSH can also be reduced to MST-SH and glutathione persulfide (GSSH) by glutathione (GSH). The GSSH is then converted to H_2_S and GSSG by the GSH. Similarly, P-SSH can interact with other proteins containing sulfydryl groups (P-SH) to form disulfide bonds between proteins (P-S-S-P) and release H_2_S (Pedre et al., 2023). Similarly, P-SH can be converted to glutathionylated proteins (P-SSG) and produce H_2_S under the action of GSSH. In addition, isothiocyanate (ITC), thiocysteine (TC), thiosulfate (TS), and allyl sulfur (AS), as major components of fertilizers and/or industrial pollutants, can release H_2_S under the action of L-Cys or GSH when they are taken up by plant roots (Nakajima et al., 2019; Yu et al., 2019; Moseler et al., 2021; Li et al., 2022; Citi et al., 2023). In addition to these, H_2_S can be released from glucose (Glc) under the influence of L-Cys, homocysteine, or methionine (Met) (**Figure 1**).

### H_2_S degradation

**Figure 2:**
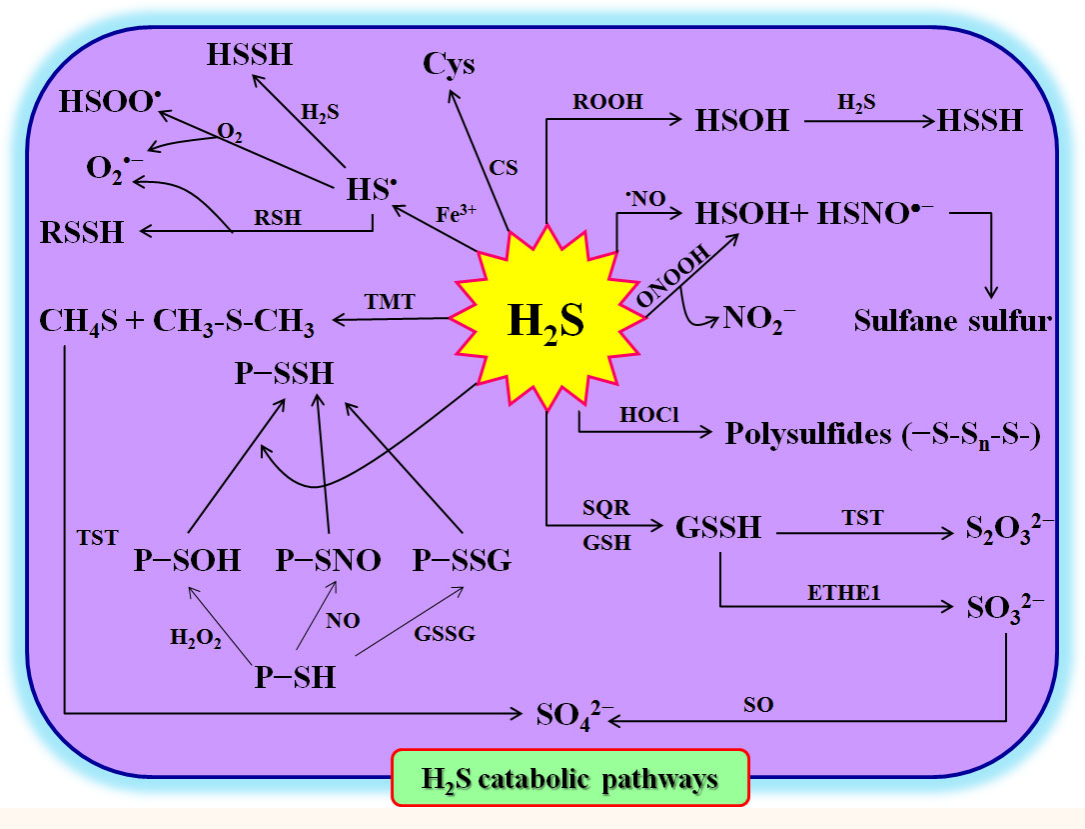
Hydrogen sulfide (H_2_S) catabolism in plants. Cys, L-cysteine; CS, cysteine synthetase; ETHE1, ethylmalonic encephalopathy 1 protein (persulfide dioxygenase); GSH, glutathione; GSSG, oxidized glutathione; GSSH, glutathione persulfide; H_2_O_2_, hydrogen peroxide; HOCl, hypochlorite; HS^•^, sulfhydryl radical; HSOH, hydrogen thioperoxide; HSOO^•^, hydroxysulfinyl radical; HSSH, hydrogen persulfide; NO, nitric oxide; ONOOH, peroxynitrous; P-SH, protein thiols; P-SNO, nitrosylated proteins; P-SOH, sulfenylated proteins; P-SSG, glutathionylated proteins; P-SSH, persulfidated proteins; RNS, reactive nitrogen species; ROOH, hydroperoxides; SO, sulfite oxidase; SQR, sulfite quinone reductase; TMT, thiol-S-methyl-transferase; TST, thiosulfate sulfur transferase; RSSH, hydropersulfides.

To maintain homeostasis, the excess H_2_S in plant cells must be rapidly scavenged, degraded, or converted. As mentioned above, in the cytosol and mitochondria, CS can utilize H_2_S and OAS to synthesize Cys, which in turn forms peptides and/or proteins (Liu et al., 2021; Yang et al., 2022; Huang and Xie, 2023) (**Figure 2**). On the other hand, in mitochondria, sulfite quinone reductase (SQR) can catalyze H_2_S and GSH to GSSH in the presence of O_2_. The GSSH can be oxidized to SO_3_^2-^ by persulfide dioxygenase (ETHE1) (Gerush and Ferenchuk, 2019; Yang et al., 2022). Subsequently, SO_3_^2-^ is further oxidized to SO_4_^2-^ and S_2_O_3_^2-^ by sulfite oxidase (SO) and Rhodanese (namely thiosulfate sulfur transferase), respectively (Gerush and Ferenchuk, 2019; Yang et al., 2022) (**Figure 2**). Similarly, H_2_S can be methylated under the catalysis of thiol-S-methyltransferase (TMT) to form methanethiol (CH_4_S) and dimethyl sulfide (CH_3_SCH_3_), and then oxidized to SO_4_^2-^ by Rhodanese (Yang et al., 2022). H_2_S can also react with ROS, reactive nitrogen species (RNS), reactive halogen species (RHS), sulfenylated proteins (P-SOH), nitrosylated proteins (P-SNO), P-SSG, and Fe^3+^ to form persulfides and/or polysulfides (Yang et al., 2022) (**Figure 2**). These persulfides and polysulfides may act as signaling molecules to further execute the physiological functions of H_2_S in plants.

## H_2_S response to HT stress

**Figure 3:**
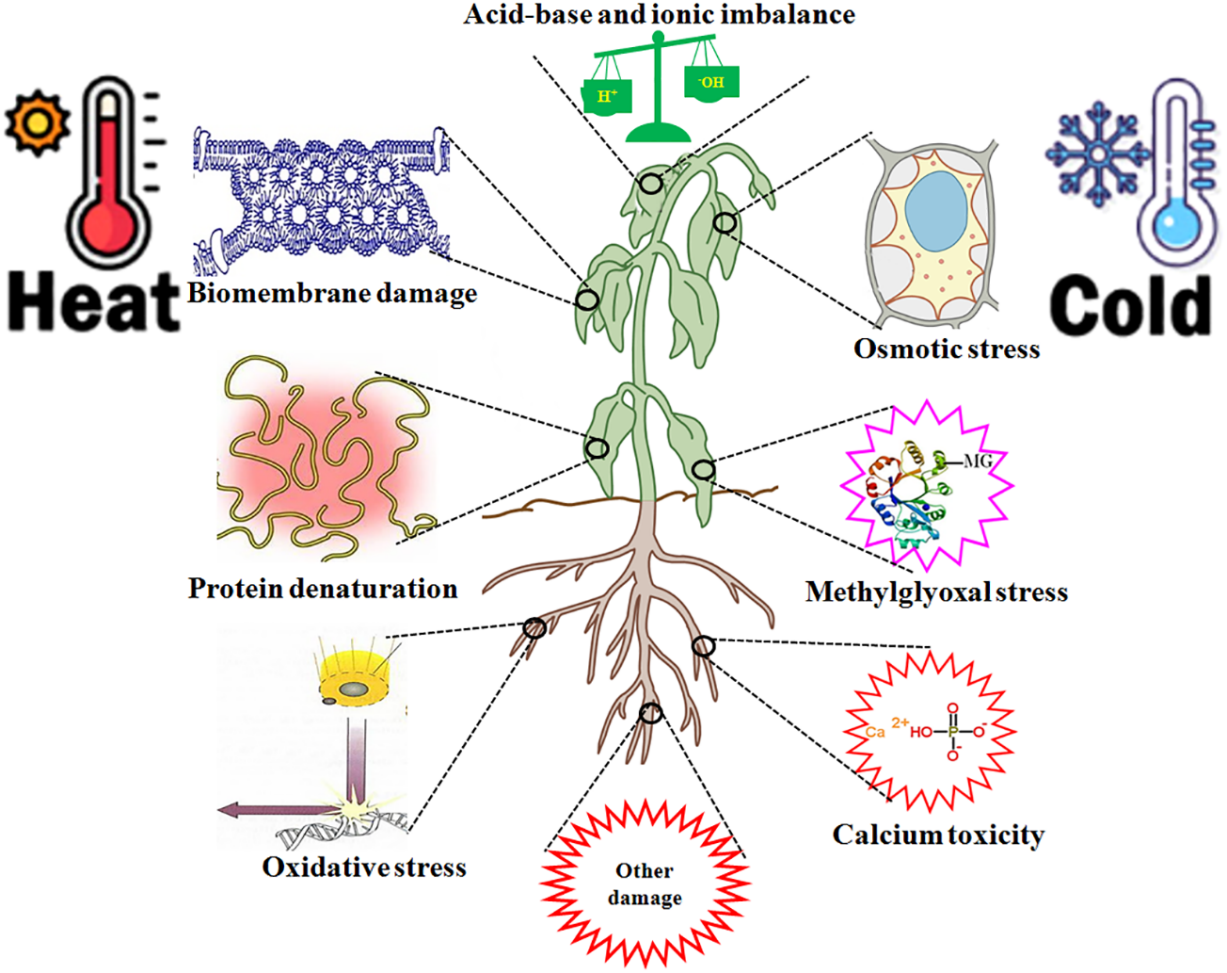
high temperature (HT) and low temperature (LT) stress injury to plants. HT and LT stress commonly cause biomembrane damage, protein denaturation, oxidative stress, osmotic stress, methylglyoxal (MG) stress, calcium overload toxicity (mainly calcium phosphate precipitation), acid-base and ion imbalance, and other damage in plants.

**Figure 4:**
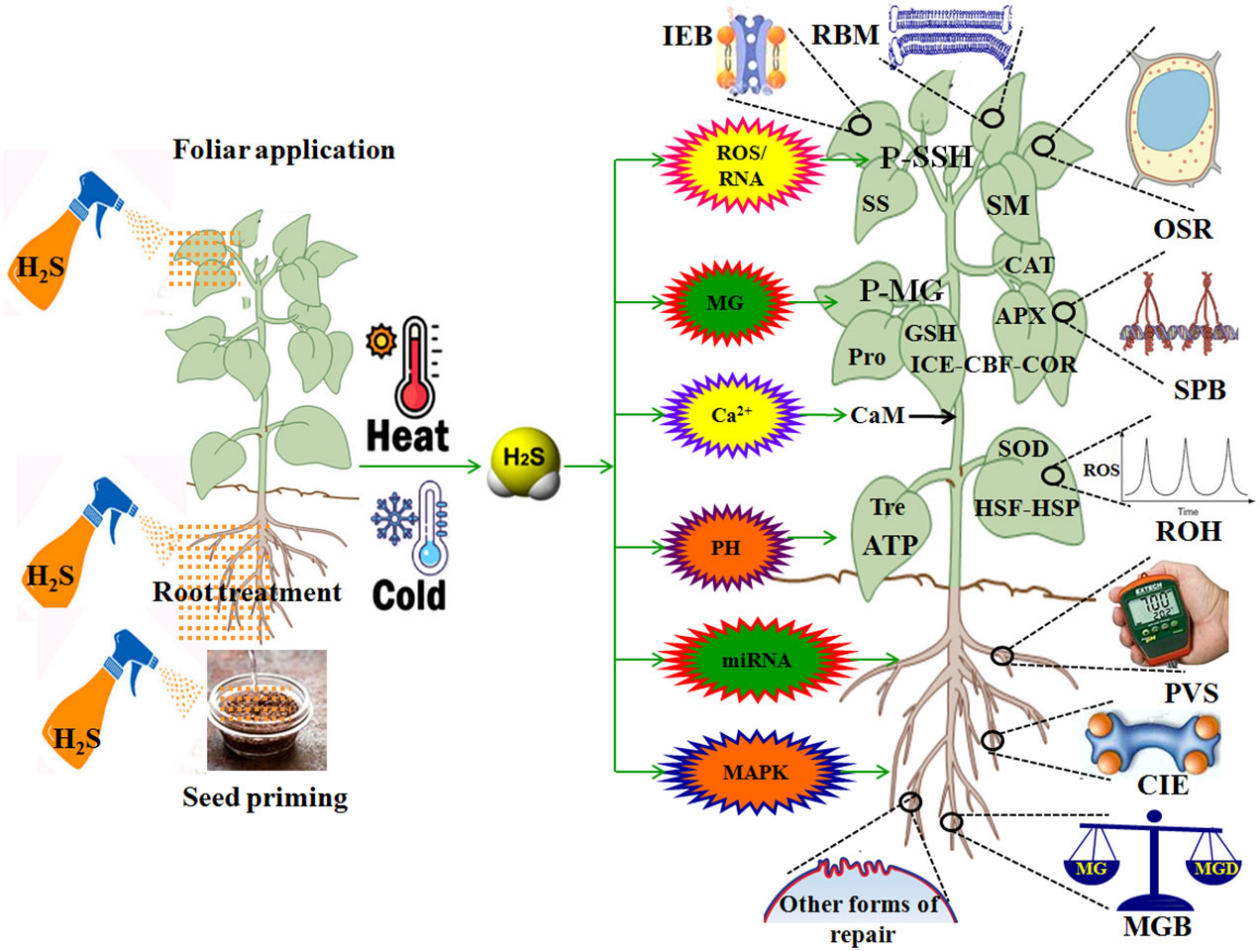
Role of hydrogen sulfide (H_2_S) in plant response to high temperature (HT) and low temperature (LT) stress. APX, ascorbate peroxidase; CBF, C-repeat binding factor; Ca^2+^, calcium ion; CaM, calmodulin; CAT, catalase; CIE, calcium ion equilibrium; COR, cold regulated proteins; DREB, dehydration response element binding proteins; GSH, glutathione; HSF, heat shock factors; HSP, heat shock proteins; ICE, inducer of CBF expression; IEB, ion equilibrium; MG, methylglyoxal; MGB, MG balance; MAPK, mitogen-activated protein kinase; miRNA, microRNA; OSR, osmoregulation; P-SSG, protein persulfidation; P-MG, protein methylglyoxalation; PVS, pH value stability; RBM, repair of biomembrane; Pro, proline; ROS, reactive oxygen species; ROH, ROS homeostasis; RNS, reactive nitrogen species; SM, secondary metabolites; SOD, superoxide dismutase; SPB, stress protein biosynthesis; SS, soluble sugars; Tre, trehalose.

HT leads to multiple injuries at the molecular, physiological, biochemical, subcellular, cellular, and whole plant body levels (Wani and Kumar, 2020; Zhang et al., 2022; Huang et al., 2023; Kan et al., 2023). HT injury is implicated in biomembrane damage, protein denaturation, Ca^2+^ overload toxicity, ion and acid imbalance, and oxidative, osmotic, and MG stress (Kosová et al., 2021; Zhao et al., 2021; Zhang et al., 2022; Zhou et al., 2022a,; Huang et al., 2023; Kan et al., 2023; Wang et al., 2023c) (**Figure 3**). Therefore, plant HT tolerance is closely related to the repair of biomembrane (RBM), stress protein biosynthesis (SPB), Ca^2+^ ion equilibrium (CIE), ROS homeostasis (ROH), osmoregulation (OSR), MG balance (MGB), ion equilibrium (IEB), and pH value stability (PVS) in plants (Zhang et al., 2022; Zhou et al., 2022a, Zhou et al., 2022b; Huang et al., 2023; Kan et al., 2023; Wang et al., 2023c) (**Figure 4**; **Table 1**).

**Table 1:** Effect of hydrogen sulfide (H_2_S) on high temperature (HT) and low temperature (LT) stress tolerance in plants.

Species	HT/LT	Effect	References
Strawberry	HT	Upregulating the gene expression of *GDH*, *GS*, *GCS*, *cAPX*, *CAT*, *Mn-SOD*, *GR*, *HSP70*, *HSP80*, *HSP90*, *DREB*, *NR*, and *PIP*; accumulating AsA and GSH; alleviating oxidative stress, nitrosative stress, and osmotic stress.	Christou et al., 2014
Maize	HT	Modulating the activity of CAT, APX, POD, GR, MDHAR, DHAR, Gly I, Gly II, and MGR, and the levels of AsA, GSH, flavonoids, carotenoids, Pro, GB, Tre, and SS by crosstalk of H_2_S with MG.	Ye et al., 2020
Maize	HT	Improving germination rate, tissue viability, and survival percentage; activating Δ^1^-pyrroline-5-carboxylate synthetase (P5CS); reducing Pro dehydrogenase (ProDH) activity; accumulating Pro; and decreasing electrolyte leakage and MDA accumulation.	Li et al., 2013
Maize	HT	Activating TPP; accumulating endogenous H_2_S and Tre; reducing electrolyte leakage and MDA levels; increasing survival rate.	Li et al., 2014a
Maize	HT	Increasing the activity of CAT, GPX, SOD, and GR, and the levels of GSH and AsA; improving survival rate in a concentration-dependent manner.	Li et al., 2014b
Tobacco	HT	Increasing survival rate, cell viability, and regrowth ability; reducing MDA accumulation by crosstalk of H_2_S with carbon monoxide (CO).	Li and Gu, 2016
Wheat and rye	LT	Accumulating Pro, SS, and anthocyanin; activating CAT and POD; reducing MDA levels; and improving survival rate.	Kolupaev et al., 2019
Pepper	LT	Inactivating SOD, peroxidase (POD), and CAT, APX, GR, MDHAR, and DHAR; upregulating the expression of *CaSOD*, *CapPOD*, *CaCAT*, *CaAPX*, *CaGR*, *CaMDHAR*, and *CaDHAR* by crosstalk of H_2_S with 5-aminolevulinic acid.	Wang et al., 2022c
Wheat	LT	Increasing the levels of amino acids, SS, GSH, and other non-protein sulfhydryl compounds.	Stuiver et al., 1992
Blueberry	LT	Accumulating chlorophylls, carotenoids, and Pro; Decreasing H_2_O_2_ and MDA levels; improving photosynthetic capacity.	Tang et al., 2020
Bermudagrass	LT	Modulating CAT, POD, GR, and GSH; accumulating Pro, SS, and sucrose; alleviating ROS burst and MDA accumulation.	Shi et al., 2013
Persimmon	LT	Increasing the activity of SOD, CAT, APX, and phenylalanine ammonia-lyase (PAL) and the levels of AsA, flavonoids, and polyphenols; reducing the accumulation of H_2_O_2_ and MDA and the activity of polygalacturonase (PG) and pectin methylesterase (PME) by crosstalk of H_2_S with -aminobutyric acid.	Niazi et al., 2021
Cucumber	LT	Upregulating the expression of GSH-associated genes (*GST Tau*, *MAAI*, *APX*, *GR*, *GS*, and *MDHAR*) and accumulating GSH; reducing H_2_O_2_, MDA, and electrolyte leakage; and increasing photosynthetic rate.	Liu et al., 2020

H_2_S improves plant HT tolerance by enhancing the antioxidant system and water homeostasis systems, biosynthesizing heat shock proteins (HSPs), and reducing biomembrane damage. In wheat (*Triticum aestivum* L.) seedlings, foliar application of sodium hydrosulfide (NaHS), an H_2_S donor, promoted HT tolerance by reducing malonaldehyde (MDA) accumulation in a concentration-dependent manner (Yang et al., 2016). H_2_S pretreatment also increased the activity of superoxide dismutase (SOD), CAT, ascorbate peroxidase (APX), and the levels of endogenous H_2_S and soluble sugars (SS). In addition, H_2_S upregulated the expression of *Fe-SOD*, *Mn-SOD*, *Cu/Zn-SOD*, *mAPX*, *tAPX*, and *CAT*, and decreased the levels of H_2_O_2_ and MDA in wheat seedlings under HT stress (Yang et al., 2016). Similarly, in strawberry (*Fragaria x ananassa* cv. ‘Camarosa’) plants, root treatment with H_2_S increased the levels of ascorbic acid (AsA) and GSH. The gene expression of AsA and GSH biosynthetic enzymes (*GDH*, *GS*, and *GCS*), antioxidant enzymes (*cAPX*, *CAT*, *Mn-SOD*, and *GR*), HSPs (*HSP70*, *HSP80*, and *HSP90*), transcription factor (*DREB*), NO biosynthesis (*NR*), and aquaporins (*PIP*) was also upregulated by H_2_S (Christou et al., 2011, Christou et al., 2014) (**Table 1**). Therefore, these molecular and physiological effects protected strawberry plants from HT damage by mitigating ROS-induced oxidative stress, NO-induced nitrosative stress, and water deficit-induced osmotic stress under HT stress conditions. Interestingly, aquaporins (AQPs) could be nitrosylated (-SNO) by NO, sulfenylated (-SOH) by H_2_O_2_, and persulfidated (-SSH) by H_2_S. These posttranslational modifications (PTMs) could change the conformation and activity of proteins, thus regulating water homeostasis in plant cells (Liu et al., 2021; Mukherjee et al., 2024). The above PTMs also indicate the crosstalk of H_2_O_2_, NO, and H_2_S in plants under HT stress conditions. In maize (*Zea mays* L.), seed priming with H_2_S increased the seed germination rate, in addition to shoot length, root length, and fresh weight of seedlings under HT stress conditions (Zhou et al., 2018). Moreover, H_2_S activated APX, SOD, CAT, glutathione reductase (GR), POD, 1-pyrroline-5-carboxylate synthetase (P5CS), proline (Pro) dehydrogenase (ProDH), ornithine aminotransferase, glycine betaine (GB) aldehyde dehydrogenase (GDH), and trehalose (Tre)-6-phosphate phosphatase (TPP) in maize seedlings (Zhou et al., 2018). The activation of these enzymes further accumulated the antioxidants AsA and GSH, and the osmolytes Pro, GB, and Tre (Zhou et al., 2018). In contrast, H_2_S-primed HT tolerance was separately enhanced by exogenous Pro, GB, and Tre, and attenuated by gabaculine, disulfiram, and sodium citrate, which are the inhibitors of osmolyte biosynthetic enzymes (Zhou et al., 2018).

H_2_S modulates HT tolerance through crosstalk with other signaling molecules. As discussed above, a large number of signaling molecules and their interactions are involved in the development of HT tolerance (Li et al., 2021; Wang et al., 2021; Zhang et al., 2022). For H_2_S-Ca^2+^ crosstalk in tobacco (*Nicotiana tabacum*) suspension-cultured cells, H_2_S pre-treatment improved HT tolerance by increasing cell viability and survival rate and decreasing electrolyte leakage and MDA accumulation (Li et al., 2012). H_2_S-induced HT tolerance was enhanced by Ca^2+^ and its ionophore A23187, whereas it was repaired by the Ca^2+^ chelator ethylene glycol-bis(β-aminoethyl-ether)-N,N,N’,N’-tetraacetic acid, the plasma membrane channel blocker La^3+^, and the calmodulin (CaM) antagonists chlorpromazine and trifluoperazine (Li et al., 2012). This study is the first to report that H_2_S interacts with Ca^2+^ in the acquisition of HT tolerance in plants. Similarly, in tobacco cells, the activity of the H_2_S-generating enzyme LCD could be activated by Ca^2+^ and CaM (Li et al., 2015a). Interestingly, H_2_S could persulfidate DES1 at Cys44 and Cys205, which in turn enhanced its activity to achieve self-amplification of H_2_S signaling (Shen et al., 2020). H_2_S was also able to regulate Ca^2+^ homeostasis in animal cells through the persulfidation of Ca^2+^ channels (Zhang et al., 2015), but this regulatory mechanism needs to be further investigated in the plant system in the future.

For H_2_S-NO crosstalk, in wheat seedlings, H_2_S and NO alone or in combination could reduce the photosynthetic suppression induced by glucose accumulation. The reduced photosynthetic suppression was closely associated with the activation of the AsA-GSH cycle and antioxidant system (CAT; SOD; APX; GR; AsA; GSH; monodehydroascorbate reductase, MDHAR; and dehydroascorbate reductase, DHAR) under HT stress conditions (Iqbal et al., 2021b). These effects were exacerbated by the NO scavenger 2-4-carboxyphenyl-4,4,5,5-tetramethylimidazoline-1-oxyl-3-oxide (cPTIO) and the H_2_S scavenger hypotaurine via the accumulation of H_2_O_2_ and MDA (Iqbal et al., 2021b). Furthermore, NO-induced HT tolerance was enhanced by H_2_S, while being reduced by hypotaurine, suggesting that H_2_S is downstream of NO to trigger the development of HT tolerance (Iqbal et al., 2021b). Similar crosstalk between H_2_S and NO was observed in the development of HT tolerance in poplar (*Populus trichocarpa*) (Cheng et al., 2018) and maize seedlings (Li et al., 2013). Interestingly, the activities of CAT1, APX1, POD5, and ROS-generating enzyme oxidase homolog protein D (RBOHD) could be separately regulated by H_2_S and NO via persulfidation (-SSH) and nitrosylation (-SNO) (Yun et al., 2011; Begara-Morales et al., 2014; Li et al., 2020; Shen et al., 2020; Terron-Camero et al., 2020). Protein persulfidation (-SSH) and nitrosylation (-SNO) could activate APX1, while inactivating CAT1 (Begara-Morales et al., 2014; Li et al., 2020; Terron-Camero et al., 2020). RBOHD could also be activated by persulfidation (-SSH), but inactivated by nitrosylation (-SNO) (Yun et al., 2011; Shen et al., 2020; Kosová et al., 2021). The persulfidation (-SSH) and nitrosylation (-SNO) of antioxidant and ROS-generating enzymes also support the crosstalk of H_2_S with NO and H_2_O_2_ in plants.

For the H_2_S-phytohormone crosstalk, in rice (*Oryza sativa* L.) cultivars, ethylene (ETH), NO, and H_2_S increased the dry weight of shoots and roots, the levels of Pro, GB, Tre, and SS, and the activity of SOD, CAT, and GR in leaves under normal and HT stress conditions. Also, the treatment improved photosynthetic parameters, such as actual, maximal, and intrinsic efficiency of PSII, in addition to photochemical quenching, non-photochemical quenching, and electron transport rate (Gautam et al., 2022). Similarly, the levels of these signaling molecules and the relative expression of *psbA*, *psbB*, *Mn-SOD*, *Fe-SOD*, *Cu/Zn-SOD*, and *APX* were upregulated by exogenous application (Gautam et al., 2022). These effects of ETH or NO were enhanced by H_2_S, while reversed by the H_2_S scavenger hypotaurine, further supporting that the ameliorative effects of ETH or NO involve H_2_S (Gautam et al., 2022). Similar signaling crosstalk of H_2_S with ETH, salicylic acid (SA), abscisic acid (ABA), and melatonin (MT) has been reported in maize seedlings (Li et al., 2015b; Wang et al., 2023b), tobacco cells (Li and Jin, 2016), and wheat seedlings (Iqbal et al., 2021a). Fortunately, the activity of 1-aminocyclopropane-1-carboxylic acid oxidase1,2 (ACO1, ACO2), *N*-acetylserotonin methyltransferase (ASMT), serotonin *N*-acetyltransferase (SNAT), abscisic acid insensitive4 (ABI4), mitogen-activated protein kinase4 (MAPK4), and SNF1-related protein kinase2.6 (SNRK2.6) could be regulated by H_2_S via protein persulfidation (-SSH) (Jia et al., 2018; Shen et al., 2020; Du et al., 2021; Zhou et al., 2021a; Wang et al., 2022b). Protein persulfidation inhibited the activity of ACO1 and ACO2, while activating ASMT, SNAT, ABI4, and MAPK4, further implying the signaling crosstalk of H_2_S with ETH, MT, and ABA in plants.

For H_2_S-ROS-NO crosstalk, H_2_S increased H_2_O_2_ and NO accumulation, in addition to NR activity in wheat plants. The increased NO was suppressed by the NR inhibitor sodium tungstate, but not by the NG-nitro-L-arginine methyl ester (NO synthase inhibitor) (Karpets et al., 2020). Similarly, NR activity and NO levels were abolished by the H_2_O_2_ scavenger dimethylthiourea and the NADPH oxidase inhibitor imidazole, whereas H_2_S-induced H_2_O_2_ levels were only weakly affected by the NO scavenger cPTIO and sodium tungstate (Karpets et al., 2020). Furthermore, H_2_S-induced HT tolerance was abolished by dimethylthiourea, imidazole, cPTIO, and tungstate (Karpets et al., 2020). This study demonstrates the signaling crosstalk of H_2_S with H_2_O_2_ and NO in the development of HT tolerance in wheat plants. A similar H_2_S-ROS-NO crosstalk was found in rice seedlings under HT stress (Gautam et al., 2022). Interestingly, in rice seedlings, NIA2 (an NR isoform), a NO-generating enzyme, could be persulfidated by H_2_S, which in turn decreased the enzyme activity, thus regulating nitrate metabolism and NO levels (Zhou et al., 2021b), further supporting the crosstalk of H_2_S with NO in plants.

## H_2_S response to LT stress

LT also results in multiple injuries in plants (Ruelland et al., 2009; Ding et al., 2019; Ritonga and Chen, 2020; Manasa et al., 2022; Gusain et al., 2023; Wen et al., 2023) (**Figure 3**), similar to that of HT (Zhang et al., 2022; Huang et al., 2023; Kan et al., 2023). Therefore, the acquisition of LT and HT tolerance in plants has some common mechanisms (**Figure 4**).

H_2_S improves LT tolerance in fruits by enhancing the ROS-scavenging system and maintaining the integrity of biomembranes and cell structures. In hawthorn (*Crateagus pinnatifida*) fruit, H_2_S fumigation increased the endogenous levels of H_2_S by activating LCD and DCD, which in turn increased the activity of SOD, CAT, APX, and phenylalanine ammonia lyase. The levels of phenols, flavonoids, anthocyanins, and AsA in fruits were also increased by H_2_S. The enhanced antioxidant system further reduced the accumulation of MDA and H_2_O_2_ under LT stress conditions, thus ameliorating LT injury in fruits (Aghdam et al., 2018). Similarly, H_2_S increased endogenous H_2_S levels by enhancing the activity of LCD, DCD, OAS-TL, and serine acetyltransferase (SAT) in peach (*Prunus persica* L.) fruits, which in turn accumulated AsA and GSH. In addition, the activity of APX, GR, CAT, and SOD was increased by H_2_S, thereby decreasing the levels of ROS (mainly H_2_O_2_ and superoxide radicals) under LT stress conditions (Wang et al., 2022a). In the same way, H_2_S decreased the activity of pectin methylesterase, polygalacturonase, β-glucosidase, carboxymethylcellulose, and β-galactosidase in peach fruits under LT stress conditions. The reduced enzyme activity inhibited the degradation of cell-wall polysaccharide fractions, thus maintaining the integrity of the cell structure (Wang et al., 2022a). The identical effects of H_2_S were found in banana (*Musa paradisiaca* L.) (Luo et al., 2015; Li et al., 2016b) and peach (Wang et al., 2023a) fruits under LT stress conditions through regulation of energy and Pro metabolism.

H_2_S enhances LT tolerance in plants through the inducer of C-repeat-binding factor (CBF) expression (ICE)−CBF−cold regulated proteins (COR) signaling pathways. In grape (*Vitis vinifera* L) seedlings, foliar application of H_2_S increased SOD activity and *VvICE1* and *VvCBF3* gene expression in leaves, and reduced superoxide anion radical and MDA levels and biomembrane damage under LT stress conditions (Fu et al., 2013). These effects were reversed by the H_2_S scavenger hypotaurine (Fu et al., 2013), suggesting that H_2_S could improve LT tolerance in grape seedlings. In the same way, H_2_S pre-treatment could improve LT tolerance of cucumber (*Cucumis sativus* L) seedlings through the CsARF5-CsDREB3 module (Zhang et al., 2021), similar to H_2_S-induced HT tolerance in strawberry (Christou et al., 2011, Christou et al., 2014). In cucumber seedlings, foliar application of H_2_S also increased the activity of APX, CAT, and POD, in addition to the levels of SS, Pro, and GSH in leaves. The enhanced ROS-scavenging system further reduced the accumulation of MDA and H_2_O_2_ and electrolyte leakage under LT stress conditions, thus improving LT tolerance of cucumber (Nasibi et al., 2020).

H_2_S modulates LT tolerance in plants through crosstalk with other signaling molecules. For H_2_S-NO-ROS crosstalk, in cucumber seedlings, H_2_S increased plasma membrane H^+^-ATPase (PMA) activity by upregulating the expression of PMA genes (*CsHA2*, *CsH4*, *CsH8*, *CsH9*, and *CsHA10*) under LT stress conditions (Janicka et al., 2018). Similarly, NO and H_2_O_2_ upregulated the expression of the gene *CsHA2*, which in turn slightly increased PMA activity in cucumber under LT stress conditions. More interestingly, NO could nitrosylate PMA, thus promoting its phosphorylation and increasing the H^+^/ATP coupling ratio under LT stress conditions (Janicka et al., 2018). This study suggests that under LT stress conditions PMA plays a key role in the development of LT tolerance in plants by maintaining PVS via the crosstalk of H_2_S with NO and H_2_O_2_. Similar effects have been observed in cucumber seedlings (Wu et al., 2022), peach fruits (Geng et al., 2019), and other plant species (Kolupaev et al., 2023) through H_2_S-H_2_O_2_ crosstalk. Interestingly, in *Arabidopsis* plants, H_2_S could persulfidate PMA, which in turn enhanced its activity, thus promoting H^+^ efflux and maintaining pH homeostasis in plant cells (Ma et al., 2023).

For the H_2_S-MAPK crosstalk, in the model plant *Arabidopsis thaliana*, H_2_S upregulated the gene expression of MPK4 under normal and LT stress conditions. The upregulation of MPK4 further activated the transcription of *ICE1*, *CBF3*, *COR15A*, and *COR15B*, which inhibited stomatal opening and alleviated LT injury (Du et al., 2017). Further studies found that H_2_S-alleviated LT tolerance was attenuated in *mpk4* mutants, but not in the upstream *mek2* and *crlk1* mutants (Du et al., 2021). More interestingly, MPK4, which has a basal persulfidation, could be further persulfidated by H_2_S, which in turn enhanced its activity nearly tenfold, whereas MEK2 was not persulfidated by H_2_S (Du et al., 2021). These data further support the fact that H_2_S alleviates LT tolerance through the MPK4 and ICE-CBF-COR signaling pathways, as discussed above.

For the H_2_S-phytohormone crosstalk, in cucumber seedlings, SA upregulated the gene expression of LCD and DCD, which in turn increased the endogenous levels of H_2_S under normal and LT stress conditions (Pan et al., 2020). This effect was blocked by paclobutrazol and 2-aminoindan-2-phosphonic acid (inhibitors of SA biosynthesis). In contrast, H_2_S and its scavenger hypotaurine and inhibitor DL-propargylglycine had no significant effect on endogenous SA levels in cucumber seedlings under normal and LT stress conditions (Pan et al., 2020). This study suggests that H_2_S plays a signaling role downstream of SA. SA and NaHS also upregulated the expression of chilling response genes (*ICE*, *CBF1*, and *COR*) and antioxidant enzymes (*SOD*, *POD*, *CAT*, *APX*, and *GR*). The upregulation of gene expression further increased enzyme activity and AsA and GSH levels. The enhanced antioxidant system reduced ROS and MDA accumulation and electrolyte leakage in cucumber seedlings under LT stress conditions, thus alleviating LT injury in cucumber (Pan et al., 2020). These data further support the fact that H_2_S enhances LT tolerance through the ICE−CBF−COR signaling pathway in grape (Fu et al., 2013), cucumber (Janicka et al., 2018; Zhang et al., 2021), and *Arabidopsis* (Du et al., 2021), as discussed above. Similarly, Zhang et al. (2020) reported that H_2_S increased the transcription and activity of flavin monooxygenase-like proteins (*YUCCA2*), thereby increasing indoleacetic acid (IAA) levels in cucumber seedlings under LT stress conditions. However, IAA treatment had no significant effect on LCD and DCD activities or H_2_S levels in cucumber seedlings under LT stress conditions (Zhang et al., 2020), indicating that H_2_S plays a signaling role in the upstream of IAA. In addition, H_2_S and IAA improved LT tolerance of cucumber seedlings by reducing electrolyte leakage and ROS accumulation. The H_2_S-induced tolerance was reduced by the IAA polar transport inhibitor (1-naphthylphthalamic acid), while the H_2_S scavenger hypotaurine had little effect on the IAA-induced tolerance (Zhang et al., 2020). These data further support the fact that IAA plays a signaling role downstream of H_2_S in plant LT tolerance through auxin response factor (ARF)−dehydration-responsive element-binding (DREB) protein signaling pathways. However, whether the ICE−CBF−COR module and plant hormone signaling proteins can be persulfidated by H_2_S needs to be further elucidated in the future.

## Conclusion and prospects

Overall, H_2_S, as a novel signaling molecule in plants, plays a pivotal role in plant growth and response to HT and LT stress through crosstalk with other signaling molecules (Shen et al., 2020; Liu et al., 2021; Wang et al., 2021; Yang et al., 2022; Mukherjee et al., 2024). As discussed above, HT and LT jointly result in biomembrane damage, protein denaturation, Ca^2+^ overload toxicity, acid-base imbalance, and oxidative, osmotic, and MG stress (**Figure 3**). To survive, plants have developed the mechanisms of tolerance to HT and LT stress by repairing the biomembrane, biosynthesizing stress proteins, enhancing the ROS-/MG-detoxification and water homeostasis systems, and maintaining Ca^2+^ and H^+^ balance (**Figure 4**). Hopefully, the tolerance mechanisms can be triggered by the application of H_2_S in the form of seed priming, foliar application, and root treatments (**Figure 4**). Briefly, under HT and LT stress conditions, the repairing of biomembrane is involved in the change of membrane lipid components, saturation, and chain length. The denatured proteins can be renatured or replaced by stress proteins, such as HSPs and cold shock proteins. Also, oxidative damage can be alleviated by detoxification systems, mainly the ROS detoxification system and the MG detoxification system. The ROS detoxification system consists of enzymes (SOD, APX, CAT, GR, DHAR, and MDHAR) and non-enzymatic antioxidants (AsA, GSH, phenols, flavonoids, and anthocyanins). The MG detoxification system includes the glyoxylase system (glyoxalase I, Gly I; Gly II; and Gly III) and the non-glyoxalase system (MG reductase, MGR; aldose/aldehyde reductase, ALR; aldo-keto reductase, AKR; and lactate dehydrogenase, LDH) (**Table 1**). Similarly, osmotic stress can be alleviated by the water homeostasis system consisting of osmolytes (Pro, GB, Tre, SS, and soluble proteins) and their metabolic enzymes (P5CS, ProDH, GDH, and TPP) and water transporters (AQPs). In addition, Ca^2+^ and H^+^ balance can be maintained by the synergistic effect of Ca^2+^–ATPase, Ca^2+^ channels, PMAs, vacuolar H^+^–ATPase, and vacuolar pyrophosphatase (Cosse and Seidel, 2021) (**Figure 4**).

Over the past decade, great progress has been made in understanding H_2_S signaling in plants from seed germination to plant HT and LT tolerance. However, several open questions need to be addressed in the future. For example, the metabolism of H_2_S, especially the non-enzymatic pathways, is not fully understood. Also, H_2_S is an emerging signaling molecule and its receptors have not been found in plants. Moreover, the exact mechanisms of H_2_S-induced HT and LT tolerance remain to be elucidated using molecular, physiological, biochemical, omics, and multi-omics approaches.

## Author contributions

Z-GL: Writing – review & editing, Writing – original draft, Conceptualization. J-RF: Writing – original draft. S-JB: Writing – original draft.
